# Respiratory Arrest and Reversible Dilated Cardiomyopathy in a Hypothyroid Dog With Chronic Collapsing Episodes Progressing to Myxedema Crisis

**DOI:** 10.1155/crve/9937245

**Published:** 2025-12-30

**Authors:** Rebecca Saunders

**Affiliations:** ^1^ Charleston Veterinary Referral Center, Charleston, South Carolina, USA

## Abstract

A 6‐year‐old male intact English Springer Spaniel was presented to the Cardiology Service at Charleston Veterinary Referral Center for evaluation of 2 months’ duration of collapsing episodes that had progressed in frequency and severity, decreased appetite, exercise intolerance, and lethargy. On presentation, the dog was depressed and dull, had a “tragic facial expression,” and a dry, thin, and brittle haircoat. A gallop sound and Grade II/VI left apical systolic murmur was ausculted with a regular rhythm but subjectively decreased pulse quality bilaterally. An echocardiogram revealed severe dilated cardiomyopathy, and a complete thyroid panel was submitted to corroborate a low total T4 that was detected on referral bloodwork from the day prior. A Holter monitor was placed to evaluate for occult ventricular arrhythmias that could explain the collapse episodes. Upon discharge from the hospital, the patient collapsed and experienced respiratory arrest. CPR was initiated and performed for approximately 2 min until spontaneous breathing occurred. Holter results (21 min in duration) at the time of arrest showed no arrhythmias and a sustained sinus rhythm. The dog was treated with heart failure medications and a loading dose of oral levothyroxine that was titrated on subsequent rechecks to achieve a total T4 within normal limits. Six months following diagnosis, his cardiac measurements had improved significantly, his cardiac medications had been reduced, and he had not experienced any additional respiratory arrest episodes since the day of his diagnosis.

## 1. Introduction

Myxedema coma or crisis is rare in animals, and when reported, it is often associated with fatality because it is difficult to recognize and treat quickly [1–3]. The mortality rate in humans is cited between 15% and 60%, and this critical clinical state is often preceded by an event that disrupts homeostatic mechanisms, such as an infection or sepsis [4, 5]. The clinical manifestations of myxedema coma in dogs are similar to those in humans, namely, altered mental status, dry hair and skin, bradycardia, and respiratory depression. Altered mental status can range from alerted awareness and mental depression to stupor to a comatose state and is caused by edema localized to the brain [4]. Respiratory depression in human and veterinary myxedema patients is characterized by a decreased ventilatory response to hypoxia and hypercarbia as well as weakness of respiratory muscles including the diaphragm [6, 7]. In humans, treatment for myxedema coma often requires mechanical ventilation for days to weeks. To the author’s knowledge, this is the first veterinary case report to describe an episode of respiratory arrest associated with a myxedema crisis in a dog that was preceded by collapse/syncopal episodes for several months. Following recovery from respiratory arrest, the dog was treated with high‐dose oral levothyroxine and did not require intravenous levothyroxine or mechanical ventilatory support.

Thyroid hormones have positive inotropic and chronotropic effects on the heart. It is well‐described in the literature that deficiency in these hormones can result in reduced left ventricular systolic function, a weak apex beat, and sinus bradycardia [8–10]. Studies have shown that thyroid supplementation in both human and canine patients can result in improved cardiac function, although left ventricular internal dimensions may be less likely to improve [8]. This case report details the moderate improvement in cardiac measurements over a 6‐month period of thyroid supplementation in a dog diagnosed with severe dilated cardiomyopathy (DCM) on presentation in a myxedema crisis.

## 2. Case Presentation

### 2.1. History

A 6‐year‐old male intact English Springer Spaniel was presented to the Cardiology Service at Charleston Veterinary Referral Center in Charleston, South Carolina, United States, for evaluation of 2 months’ duration of collapsing episodes that had progressed in frequency and severity, hyporexia progressing to anorexia, exercise intolerance, and lethargy. The owner described the dog’s collapse as episodes in which he would suddenly shift from sitting in a sternal position to suddenly lying laterally. He would become nonresponsive and appeared to stop breathing during these episodes. For 3 days prior to presentation, the owner had reported that the episodes happened multiple times a day and that she had noticed the dog’s gums would become cyanotic during the episodes. A total T4 submitted by the referring veterinarian the day prior to presentation to the Cardiology Service was low (0.5 *μ*g/dL, reference range 0.8–3.5 *μ*g/dL).

### 2.2. Physical Exam and Preliminary Investigation

**Figure 1:**
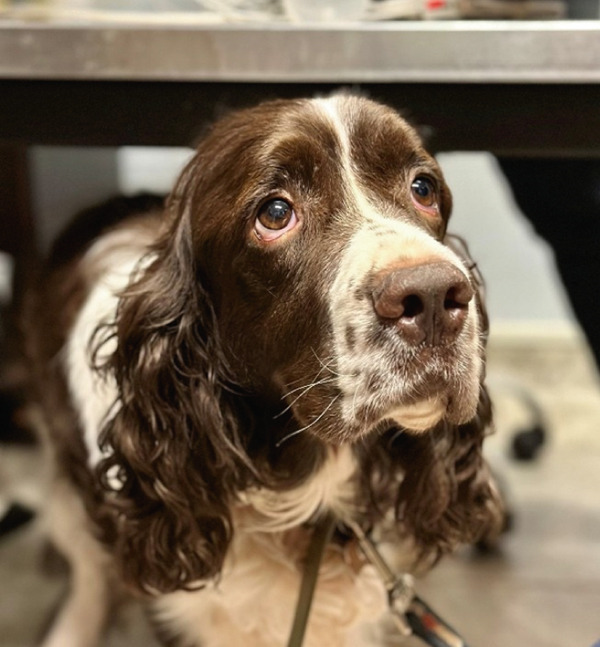
Tragic facial expression of the patient described in this report.

Upon presentation to Charleston Veterinary Referral Center, the dog weighed 25.6 kg with a body condition score of 6/9 (ideal: 5/9), had a dry and brittle haircoat, “tragic facial expression,” and had a depressed and dull mentation (Figure 1). Temperature (100.1 F) and heart rate (150 beats per minute) were within normal limits. Mucous membranes were pale, and the patient appeared weak, unwilling to stand or sit upright in favor of lying down. There was moderate tachypnea (60 breaths/minute) noted, and a gallop sound ausculted in addition to a Grade II/VI left apical systolic murmur.

**Figure 3:**
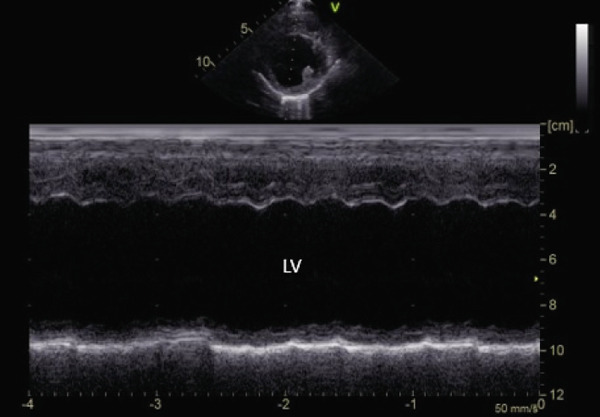
M‐mode right parasternal short‐axis echocardiographic image demonstrating poor global myocardial systolic function and severe left ventricular dilation in a hypothyroid dog imaged during myxedema crisis. Ejection fraction (Teicholz) was 26% (normal: 33%–46%) [11].

An echocardiogram was performed that revealed severe left ventricular and left atrial dilation (Figure 2a,b and Table 1), marked systolic dysfunction (Figure 3 and Table 1), mild right ventricular and atrial dilation, moderate mitral regurgitation, and mild to moderate tricuspid regurgitation. No pleural effusion or ascites was noted. These findings were consistent with a diagnosis of severe DCM. It was likely that the disease was secondary to the patient’s hypothyroid state (differential diagnosis: primary DCM). The blood pressure obtained via Doppler measurement was 80 mmHg (normal range: 100–160 mmHg). Bloodwork was obtained for evaluation of T4 by equilibrium dialysis (ED) and thyroid‐stimulating hormone (TSH). Finally, a Holter monitor was placed to evaluate for occult ventricular arrhythmias that could explain the patient’s collapsing episodes.

Figure 2(a) Right parasternal long‐axis echocardiographic image demonstrating severe left ventricular and left atrial dilation in a hypothyroid dog imaged during myxedema crisis. (b) Right parasternal short‐axis echocardiographic image demonstrating severe left ventricular dilation and thinned appearance to the left ventricular walls in a hypothyroid dog imaged during myxedema crisis. The interventricular septum measured 6.92 mm in diastole (normal indexed to body weight: 9.3–10.2 mm); the left ventricular free wall measured 6.92 mm in diastole (normal indexed to body weight: 7.5–8.2 mm) [11].

**Figure:**
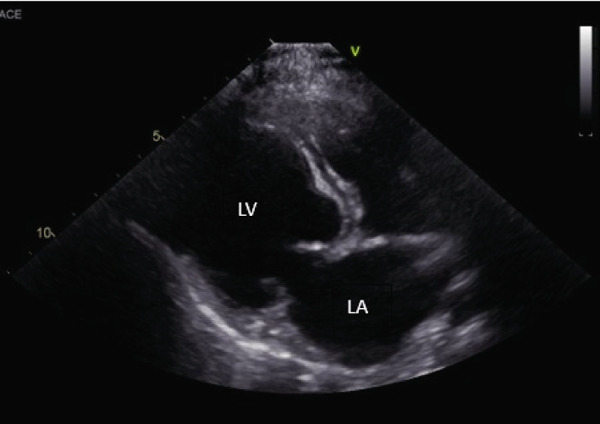
(a)

**Figure:**
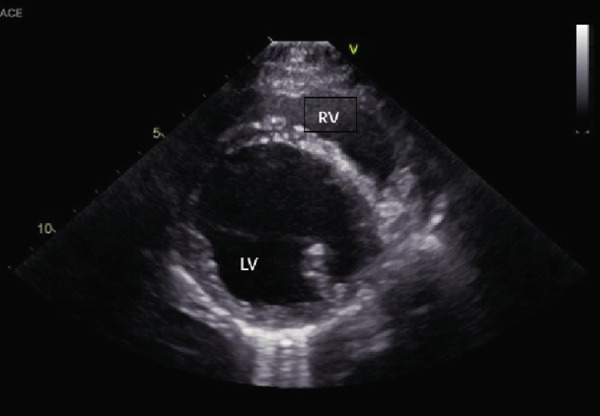
(b)

**Table 1:** Comparison of echocardiographic variables of the dog described in this report over a 6‐month time period.

**Patient’s echocardiographic parameter**	**Cardiac measurements at time of diagnosis (no thyroid supplementation)**	**Cardiac measurements 10 weeks after diagnosis (thyroid supplementation, TT4 WNL)**	**Cardiac measurements 6 months after diagnosis (thyroid supplementation, TT4 WNL)**	**Normal parameter values indexed to patient’s body weight [** **11** **]**
LVIDd (cm)	5.784	4.438 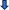	4.720 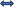	3.75–4.03
LVIDs (cm)	5.070	3.255 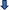	3.710 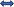	2.30–2.50
LA (cm)	4.2	2.6 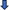	3.0 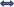	2.24–2.41
LA/Ao (cm)	2.27	1.33 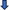	1.40 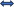	1.0–1.3
FS (%)	12	27 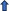	26 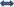	35–45

Abbreviations: Ao, aorta diameter; FS, fractional shortening; LA, left atrium; LVIDd, left ventricular internal dimension at diastole; LVIDs, left ventricular internal dimension at systole.

**Figure 4:**
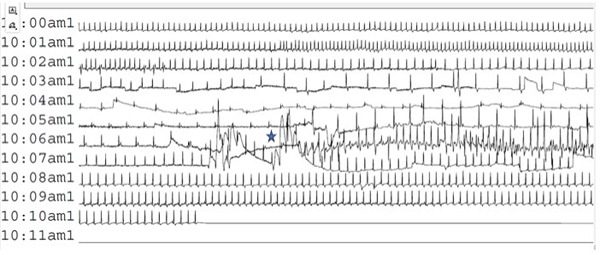
Holter monitor recording excerpt taken during an episode of respiratory arrest in a hypothyroid dog during myxedema crisis. The star indicates when chest compressions were initiated during CPR.

While discharging the dog with the Holter monitor in place, the patient acutely fell into lateral recumbency from a sternal position, became unresponsive, stopped breathing, and became cyanotic. The dog was then carried to the triage area, and CPR was initiated when respiratory arrest was confirmed following patient intubation and a capnography reading (ETCO2) of zero. Chest compressions were performed for 2 min while an IV catheter and monitoring were placed. Before cardiopulmonary arrest drugs could be administered, the patient began spontaneously breathing, and CPR was discontinued. The Holter monitor was then removed and submitted for STAT reading. Holter results clearly showed when CPR had been initiated (Figure 4), in addition to revealing that the patient’s heart rhythm never stopped during the entire collapse and respiratory arrest event. No significant ventricular arrhythmias or pauses were noted on the 21‐min recording.

### 2.3. Case Management

Given (1) the low total T4 on referral bloodwork, (2) the patient’s physical exam findings, (3) echocardiographic findings consistent with severe DCM, and (4) the patient’s acutely critical state associated with owner‐reported and now directly documented episodic respiratory arrest, an oral loading dose of levothyroxine was administered (0.04 mg/kg), in addition to an oral dose of Vetmedin (R) (0.3 mg/kg). The patient was monitored on continuous ECG recording for 3 h with no arrhythmias noted. The owner declined further hospitalization and elected to take the patient home for continued monitoring. The patient was discharged with the following medications: furosemide (1.6 mg/kg PO q 12 h), benazepril (0.4 mg/kg PO q 12 h), Vetmedin (R) (0.3 mg/kg PO q 12 h), and levothyroxine (0.02 mg/kg PO q 12 h). A follow‐up phone call later that afternoon revealed that the dog had experienced an additional cyanotic collapse episode when the patient arrived home but had since recovered. The owner declined to bring the dog back in for longer hospitalization and monitoring.

### 2.4. Emergent Re‐Presentation

Five days after initial presentation, the dog was re‐presented to the Emergency Service at Charleston Veterinary Referral Center for an 8‐h history of lethargy and anorexia. On presentation, the dog had an irregular cardiac arrhythmia that was diagnosed as atrial fibrillation with a ventricular response rate of 240 beats/minute based on a diagnostic ECG recording. The dog’s temperature on presentation was mildly febrile at 103.6, and he was moderately tachypneic (60 breaths/minute). Focused Assessment with Sonography in Trauma (FAST) scan of the thorax and abdomen revealed no pleural, pericardial, or peritoneal effusions. Thoracic radiographs revealed very mild enlargement of the left atrium and concern for developing bronchopneumonia. The patient was hospitalized on continued oral medications as listed above, in addition to Cerenia (R) (1 mg/kg IV q 24 h) for potential nausea causing anorexia, Unasyn (30 mg/kg IV q 8 h) for concern for developing bronchopneumonia, diltiazem SR (2.3 mg/kg PO q 12 h), and trazodone for concern for developing bronchopneumonia, and trazodone (50 mg PO once) for anxiety. The patient’s rhythm converted to a sinus rhythm at a rate of 115 beats/minute after a single oral dose of diltiazem (2.5 mg/kg), and the patient remained in sinus rhythm for the remainder of hospitalization.

On physical examination the following day, the patient had a Grade III/VI left apical systolic murmur, a sinus rhythm with occasional ventricular bigeminy noted, a normal respiratory rate and effort, and a brighter mentation. Bloodwork was submitted for a comprehensive tick panel to screen for infectious tick‐borne diseases, and the patient was discharged with the following medications: Vetmedin (R) (0.3 mg/kg PO q 12 h), furosemide (1.6 mg/kg PO q 12 h), levothyroxine (0.02 mg/kg PO q 12 h), Cerenia(R) (2.4 mg/kg PO q 24 h), omeprazole (1 mg/kg PO q 12 h), ondansetron (0.6 mg/kg PO q 12 h), and acetaminophen (12.7 mg/kg PO q 12 h) for possible pain/discomfort from pulmonary contusions secondary to chest compressions performed the week prior. Antibiotics were discontinued due to regurgitation noted after Unasyn (R) was administered in the hospital, and the more likely suspicion that the pulmonary infiltrate was cardiogenic pulmonary edema and not emerging bronchopneumonia. The ACE inhibitor was also temporarily discontinued due to the patient’s variable appetite and concern for inducing azotemia or acute kidney injury.

### 2.5. Additional Diagnostics and Initial Follow‐Up

The patient’s thyroid panel from initial presentation confirmed the diagnosis of hypothyroidism with a free T4 of 3.4 pmol/L (normal range: 8–40) and TSH of 1.19 ng/mL (normal range: 0–0.6). The North Carolina State University tick panel results were negative for all infectious diseases tested. A total T4 evaluated 2 weeks after initiation of levothyroxine was > 10 *μ*g/dL (normal range: 1–4), so the patient’s levothyroxine dose was reduced to 0.01 mg/kg PO BID. At that time, the owner reported no additional episodes of collapse, improved energy, and increased urination. Two weeks later, both total T4 and free T4 by ED were within normal limits (2.6, normal range: 0.8–3.5; 32.4, normal range: 8–40), respectively. The dog was clinically doing well at that time.

### 2.6. Case Outcome

Six weeks after initial diagnosis, a Holter monitor was placed to obtain a full 24‐h recording in the dog’s home environment to determine if occult ventricular arrhythmias were present that would warrant discontinuing diltiazem in favor of a ventricular antiarrhythmic medication. The dog’s Holter showed very mild ventricular arrhythmias characterized by 640 ventricular premature complexes (< 1% of total beats), all isolated except for one ventricular couplet. The results of this Holter further supported a nonarrhythmogenic cause of the patient’s historical collapse episodes. The patient was continued on diltiazem 60 mg once daily.

**Figure 6:**
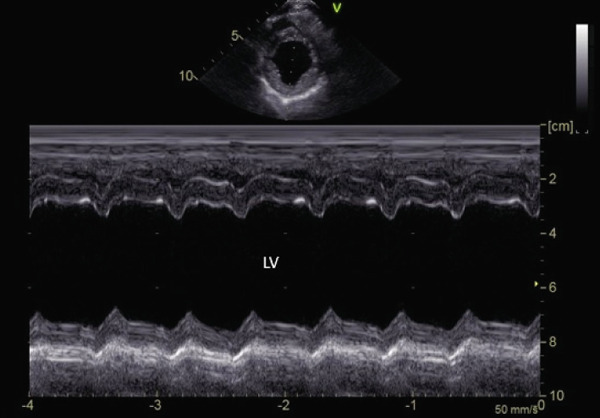
M‐mode right parasternal short‐axis echocardiographic image demonstrating significantly improved global myocardial systolic function and reduced left ventricular dilation in the same dog after receiving levothyroxine for 10 weeks. The ejection fraction (Teicholz) was 52% (normal: 33%–46%) [11].

Ten weeks after initial diagnosis, the patient was presented for a recheck echocardiogram. Results are detailed in Table 1. Left ventricular internal dimensions and left atrial dimensions were all markedly reduced in size (Figure 5a,b and Table 1). Estimates of systolic function were moderately to markedly improved (Figure 6). All medications were continued as previously prescribed, and benazepril was restarted (0.5 mg/kg PO q 12 h). Four months later (6 months after diagnosis), an echocardiogram was again performed and revealed stable cardiac measurements. A 5‐min ECG showed a normal sinus rhythm, and total T4 was within normal limits (1.5, normal range: 0.8–3.5). Furosemide was reduced to 1.3 mg/kg PO q 12 h, and diltiazem and benazepril were discontinued. Vetmedin (R) (0.3 mg/kg PO q 12 h) and levothyroxine (0.01 mg/kg PO q 12 h) were continued. At the time of this writing, 11 months after diagnosis, the owner reports that the dog has had no additional respiratory arrest episodes, is very active, has a strong appetite, and a much shinier, healthier haircoat.

Figure 5(a) Right parasternal long‐axis echocardiographic image demonstrating mild–moderate left ventricular dilation (improved from severe) and mild left atrial dilation (improved from severe) in the same dog after receiving levothyroxine for 10 weeks. (b) Right parasternal short‐axis echocardiographic image demonstrating mild–moderate left ventricular dilation and normal left ventricular wall thickness in the same dog after receiving levothyroxine for 10 weeks. The interventricular septum measured 9.37 mm in diastole (normal indexed to body weight: 9.3–10.2 mm); the left ventricular free wall measured 8.95 mm in diastole (normal indexed to body weight: 7.5–8.2 mm) [11].

**Figure:**
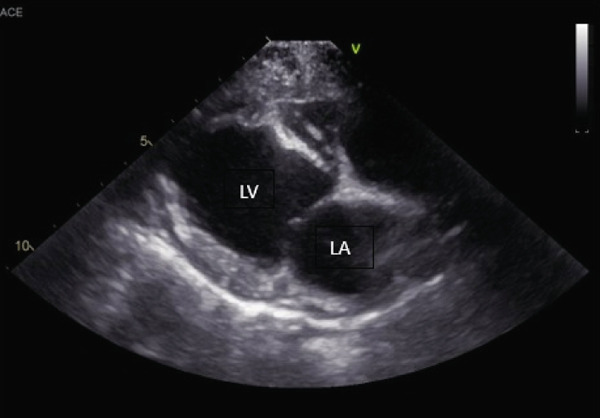
(a)

**Figure:**
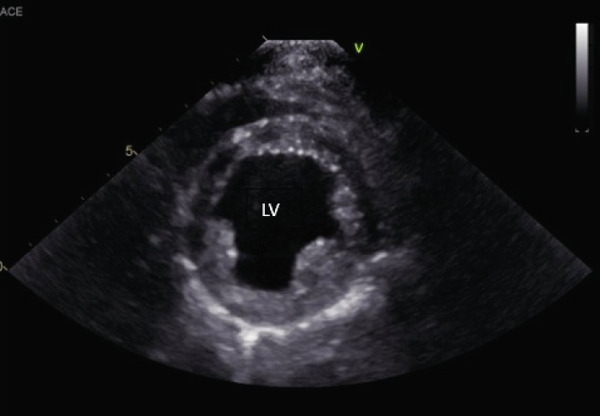
(b)

## 3. Discussion

### 3.1. Myxedema Crisis and Respiratory Compromise

Myxedema coma is a rare, life‐threatening manifestation of severe, chronic hypothyroidism and is considered an endocrine crisis in both humans and veterinary patients. In addition to a low total T4 on referral bloodwork, other biochemical abnormalities were noted in this canine patient supportive of a diagnosis of hypothyroidism. Hyponatremia (the dog described in this report: 138 mEq/L, normal range: 139–154) is a result of decreased renal blood flow and decreased glomerular filtration rate, resulting in the reduced ability to excrete water and excessive antidiuretic hormone secretion [3]. Hyponatremia contributes to mental depression that is exacerbated by hypercarbia [3, 12]. Mild anemia was also documented in this patient (30%, normal range: 36%–60%) and is due to decreased erythropoiesis and lower erythropoietin levels [13].

**Figure 7:**
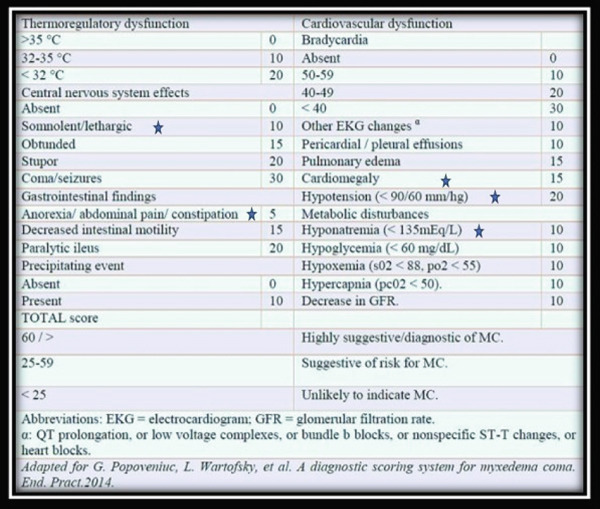
Diagnostic scoring system for myxedema coma in humans. Stars indicate the clinical findings identified in the dog described in this case report.

In humans, the incidence of myxedema coma is not well‐documented in the USA, and symptoms are vague and can affect many body systems. Therefore, recognition of the disease presents a diagnostic challenge. To aid in the rapid identification of this dangerous condition, a clinical tool was developed in 2014 for scoring a suspected myxedema coma patient based on clinical features of multiorgan dysfunction (Figure 7). If this scoring system were extrapolated to the dog in this case report, his myxedema score would be 60, which is highly suggestive of myxedema crisis.

Collapse associated with episodic respiratory arrest in a myxedema crisis patient has not been reported in the veterinary literature to the author’s knowledge. A case report in 2004 described progressive respiratory depression in a Cocker Spaniel being treated for myxedema coma; the patient was ultimately humanely euthanized [3]. Similarly, a case report in 2021 described hospitalized treatment of a mixed‐breed dog in myxedema crisis who ultimately became agonal and respiratory arrested [1]. It was fortuitous that the patient described in this report was wearing a Holter monitor during this episode, as it allowed us to strongly support that the episodes the owner had been witnessing were not arrhythmogenic in origin and were likely a result of respiratory compromise and possible pulmonary arrest.

### 3.2. Hypothyroidism and DCM

Studies have shown that naturally occurring hypothyroid humans and dogs have significantly increased left ventricular internal dimensions and decreased fractional shortening and ejection fraction [8]. In the myocardium, thyroid hormone controls myosin enzyme production, sarcolemma calcium ATPase activity, sodium–potassium ATPase activity, and calcium channel activity. These changes, along with changes in the peripheral circulation, including increased systemic vascular resistance, result in decreased cardiac output, impaired cardiac function, and impaired relaxation [8, 14].

Some markers of hypothyroidism at the tissue level change slowly following levothyroxine supplementation with normalization potentially taking months, but complete resolution of cardiac dilation and systolic dysfunction has been reported in dogs [8, 14, 15]. If structural or functional cardiac changes do persist despite the establishment of a euthyroid state for several months, the cause is believed to be multifactorial. Decreased myocardial cellular metabolism associated with severe hypothyroidism can contribute to permanent cardiac dysfunction secondary to atherosclerosis and myocardial fibrosis [14]. In humans, myxedema of the heart (as confirmed on endomyocardial biopsy) can lead to irreversible myocardial damage and fibrosis [16]. Although this patient’s cardiac measurements have not yet normalized 6 months after starting supplementation, his left ventricular internal dimensions and left atrial dimensions have significantly reduced in size (by an average of 30% and 38%, respectively), and fractional shortening is significantly improved (by 55%). Residual systolic dysfunction may be associated with myocardial infiltration of mucopolysaccharides, coronary arterial atherosclerosis, or both, but this would require either an antemortem endomyocardial biopsy or postmortem necropsy for definitive diagnosis [14, 17].

Finally, it is relevant to note that the dog described in this report re‐presented 5 days after initial diagnosis with recurrence of lethargy and decreased appetite, presumptively caused by the sudden onset of atrial fibrillation that was not present on initial presentation. As previously discussed, primary hypothyroidism depresses contractility and decreases the cardiac output of the left ventricle in both dogs and humans. Despite this impaired function, however, the left ventricle is usually able to meet the decreased metabolic demands of the body in primary hypothyroidism. However, in humans with comorbidities such as hypertension or coronary artery disease, congestive heart failure may develop due to the additional demands (or limitations) placed on ventricular function with these concurrent diseases. Similarly, comorbidities in dogs, such as mitral insufficiency or DCM, may also burden the already‐compromised left ventricular function, resulting in subsequent atrial dilatation. This creates the substrate and environment for the inception and maintenance of atrial fibrillation. Additionally, an increase in peripheral resistance (afterload) in primary hypothyroidism, in combination with decreased myocardial function, will increase the ventricular end diastolic volume and wall tension (preload) in the ventricle, resulting in further dilatation of the left atrium, once again spurring the onset of atrial fibrillation [18, 19]. The patient in this report proved extremely responsive to calcium channel blockade and converted to a sinus rhythm with a single dose of diltiazem. He was maintained on once‐daily diltiazem until 6 months after diagnosis when this medication was discontinued because no recurrent supraventricular arrhythmias had been documented on ECG and Holter evaluations, and the patient’s left atrium had reduced to mild dilatation.

Limitations of this report include the ability to characterize each collapse episode with certainty. Although the patient’s events increased in frequency and severity culminating in a documented episode of respiratory arrest, it is possible that the earlier episodes differed in nature and were episodes of cardiogenic syncope secondary to DCM or occult arrhythmias. Because continuous monitoring (e.g., Holter or Event recording) was not available during each episode and the descriptions of prior events relied solely on owner observation, it cannot be determined whether the earlier collapse episodes represented paroxysmal respiratory arrest. Additionally, the diagnosis of myxedema coma or crisis in veterinary medicine is predominantly presumptive, not definitive. It is based on signalment, clinical signs, and supporting clinicopathological features in the face of a low total T4. Finally, the dog in this report was treated with both thyroid supplementation and cardiac medications at the time of diagnosis of myxedema crisis and responded favorably to dual treatment, further confounding whether the historical collapse episodes were a symptom of DCM or a symptom of progressing severity of hypothyroidism.

Myxedema coma or crisis is a rare complication of hypothyroidism that is challenging to recognize in clinical practice and is associated with a high mortality rate [2]. This report describes a unique case in which a dog with a presenting complaint of frequent collapse was ultimately diagnosed with episodic respiratory arrest secondary to a myxedema crisis state. Treatment with a loading dose of oral levothyroxine resulted in the resolution of these episodes within 24 h. Additionally, this report documents substantial improvement in the dog’s cardiac structure and function measurements as the patient improved from severe to mild DCM within 6 months of supplementation.

## Conflicts of Interest

The author declares no conflicts of interest.

## Funding

No funding was received for this manuscript.

## Data Availability

The data that support the findings of this study are available from the corresponding author upon reasonable request.
